# Insights into urticaria in pediatric and adult populations and its management with fexofenadine hydrochloride

**DOI:** 10.1186/s13223-022-00677-z

**Published:** 2022-05-13

**Authors:** Ignacio J. Ansotegui, Jonathan A. Bernstein, Giorgio W. Canonica, Sandra N. Gonzalez-Diaz, Bryan L. Martin, Mario Morais-Almeida, Margarita Murrieta-Aguttes, Mario Sanchez Borges

**Affiliations:** 1 Department of Allergy and Immunology, Hospital Quironsalud Bizkaia, Leioa-Unbe Errepidea, 33 Bis, Erandio, 48950 Bilbao, Spain; 2 Department of Internal Medicine, Division of Allergy and Immunology, University of Cincinnati, Cincinnati, OH USA; 3 Personalized Medicine, Asthma and Allergy, Humanitas University and Research Hospital, Rozzano, Milan Italy; 4 Regional Center for Allergy and Clinical Immunology, Universidad Autónoma de Nuevo León, Monterrey, México; 5 Medicine and Pediatrics, The Ohio State University in Columbus, Columbus, OH USA; 6 Allergy Center, CUF Descobertas Hospital, CUF Academic and Research Medical Center, Lisbon, Portugal; 7 Sanofi, Scientific Innovation, Gentilly, France; 8 Allergy and Clinical Immunology Department, Centro Médico Docente La Trinidad, Caracas, Venezuela

**Keywords:** Urticaria, Fexofenadine hydrochloride, Clinical guidelines, Second-generation antihistamines, Pediatric, Adults

## Abstract

**Objective:**

The present narrative review provides a comprehensive update of the current knowledge on urticaria, both in adult and pediatric populations, and on the safety and efficacy of fexofenadine hydrochloride (HCl) as a treatment option.

**Data source:**

A literature search was conducted on Embase and Medline.

**Study selection:**

Clinical studies published in English and published between 1999 and 2020 were selected.

**Results:**

Although the exact pathogenesis of urticaria is not fully understood, multiple pathways of mast cell activation are discussed to explain the existence of phenotypically different clinical manifestations of urticaria. An overview of the worldwide prevalence of chronic urticaria, including disease burden and patient’s quality of life is provided. The impact of urticaria on patient’s life differs on the basis of whether its form is acute or chronic, but pharmacological approaches are most often needed to control the disabling symptoms. A summary of the current management of urticaria recommended by different guidelines across countries (Global; European; American; Australian; Asian; Japanese) is presented. Non-sedating, second-generation H_1_-antihistamines are the preferred choice of treatment across several guidelines worldwide. Herein, the efficacy and safety of fexofenadine HCl, a representative second-generation H_1_-antihistamine approved for the treatment of urticaria, is discussed. The occurrence of urticaria manifestations in COVID-19 patients is also briefly presented.

**Conclusion:**

The burden of acute and chronic urticaria is high for patients. Second generation anti-histamines such as fexofenadine HCl can help managing the symptoms.

## Introduction

Urticaria and angioedema are frequent conditions among the general population requiring medical consultation, diagnostic testing, and implementation of preventive and pharmacological approaches to control the disabling symptoms suffered by the patient [1]. Although there have been important advancements in the recognition of pathophysiological pathways leading to the development of wheals and angioedema, there are still many knowledge gaps that require further investigation [1]. Second-generation antihistamines are the first-line pharmacological approach to resolve urticaria’ symptoms [1]. Fexofenadine hydrochloride (HCl) is one of the second-generation antihistamines available on the market and a valid option for the treatment of urticaria in adult and pediatric populations.

The present article provides an updated review of the current knowledge on urticaria with a focus on the efficacy and safety of fexofenadine hydrochloride (HCl). A literature search was conducted on Embase and Medline. Eligible articles included information on urticaria prevalence, classification, pathogenesis, etiology, disease burden, impact on patient’s quality of life (QoL), diagnostic approach and practical management, and clinical studies on the safety and/or efficacy of fexofenadine HCl. The search was limited to articles published in English between 1999 and 2020 and a total of 180 publications were reviewed. Following the outbreak of the coronavirus disease 2019 (COVID-19) and the occurrence of urticaria manifestations in COVID-19 patients, available studies on this matter are also briefly discussed.

## Prevalence and clinical features of urticaria

Urticaria is a common disease worldwide that can occur at any age. An estimated 20% of adults have experienced an episode of acute urticaria at least once in their life [1], with up to 1.4% experiencing a chronic form [2]. A recent investigation of the world prevalence of chronic urticaria reported that it was higher in South America, followed by Asia, Europe, and North America (there is currently no data available from Africa) [2].

Urticaria is defined as the occurrence of wheals (hives), angioedema, or both. Wheals are erythematous and superficial edema of the skin associated with severe itching (pruritus) or burning sensation which can be generalized over the entire body or localized to specific areas [3]. Wheals affect the superficial skin layers (dermis), ranging from a few millimeters to several centimeters which usually disappear within 24 h [3, 4]. Angioedema is a localized edema that involves the deeper dermis and subcutaneous tissues and presents as painful or burning rather than itchy [3]. Compared with wheals, angioedema develops slowly and usually has a longer duration, lasting up to few days before remitting [3]. Approximately 50% of urticaria sufferers present exclusively with wheals, 40% present with wheals plus angioedema, and 10% exhibit angioedema alone [3].

Urticaria and angioedema should not be confused with other skin manifestations such as auto-inflammatory diseases, urticarial vasculitis, bradykinin-mediated adverse events, drug reactions, anaphylaxis, urticaria pigmentosa, ectoparasitosis, contact dermatitis, and autoimmune bullous diseases [1].

## Classification of urticaria

The classification of urticaria, valid for both adult and pediatric populations, is based on the duration and cause of symptoms. There are several subtypes of urticaria, a summary of which and corresponding etiology is presented in Table 1. Depending on whether the skin lesions appear spontaneously or are induced by a specific trigger, urticaria can be further classified as spontaneous or inducible [1, 5]. In the most recent classification, the term ‘inducible” replaced “physical” to account for other types of triggers (e.g. cholinergic or adrenergic) rather than just physical ones [3]. Similarly, the term ‘idiopathic’ has been replaced by “spontaneous” to highlight the lack of a specific trigger and the unknown mechanism of mast cell activation [3, 6]. Unfortunately, cross-reactivity during allergy testing can make determining the primary causative factor difficult, so many cases remain ‘idiopathic’ [7].

**Table 1:** Classification of urticaria [1, 9]

Type	Subtype	Cause
**Spontaneous urticaria**		
	Acute urticaria	
	Chronic urticaria	Infections (bacterial, viral, parasitic, fungal)
		Food and additives
		Drugs (e.g. NSAIDs; ACE)
		Emotional stress
		Autoimmune disorders
**Inducible urticaria**		
Physical	Dermographism	Mechanical shearing force (rubbing or scratching)
	Cold urticaria	Cold air; cold liquid; or cold solid
	Delayed pressure urticaria	Vertical pressure
	Heat urticaria	Local heat exposure
	Solar urticaria	Ultraviolet or visible light
	Vibratory angioedema	Vibratory forces
Other types	Aquagenic urticaria	Water; sweat; lacrimation
	Cholinergic urticaria	Increasing core body temperature (e.g. exercise; fever)
	Contact urticaria	Contact (e.g. foods; plant components; latex; drugs, cosmetics; textiles)

*ACE* angiotensin-converting enzyme, *NSAIDs* non-steroidal anti-inflammatory drugs

### Acute urticaria

Acute urticaria, while more prevalent in females, is less common in adults and is predominately considered a childhood affliction [6]. Acute urticaria is often self-limiting, with episodes resolving within six weeks [1]. Acute urticaria has been associated with upper respiratory tract infections (39.5%), drugs, such as non-steroidal anti-inflammatory drugs (NSAIDs) (9.2%), and food intolerance (0.9%); however, the majority of cases are considered spontanoues [6, 8]. This type of urticaria is initially treated in a primary care setting and diagnosis relies on a detailed history of traits, duration and possible causative factors, along with evaluation of lesions to ensure absence of inflammatory urticaria [6]. Although acute urticaria is rarely associated with immunoglobulin E (IgE)-mediated events, cases triggered by foods, drugs or external agents are often IgE-dependent [7, 8].

### Chronic urticaria

Chronic urticaria is defined by recurrent wheals, with or without angioedema, that persist for six weeks or longer [2]. The identification rate of a specific cause of chronic urticaria ranges between 15 and 20% [9] but, similar to acute urticaria, infections, inducible factors (e.g. physical), food and drugs are the most common triggers [1, 4, 9].

Spontaneous urticaria accounts for approximately 50–75% of chronic urticaria cases [6], and is considered a multifactorial pathology, involving both endogenous and exogenous factors [6]. An estimated 50% of cases display an autoimmune etiology, where IgG autoantibodies directed against IgE (IgG anti-IgE) are present on mast cells and basophils [7].

Chronic inducible urticaria accounts for approximately 20–35% of cases and is commonly induced by cold, heat, dermographism, pressure, vibration, sunlight or water [7]. Regardless of the cause, symptoms occur only after adequate stimulus and remain localized at the stimulus site, usually resolving within an hour. Diagnosis often involves a provocation test, but it is not uncommon to identify more than one inducible form in a single patient [6]. In chronic inducible urticaria, systemic involvement may vary between the different subtypes (e.g. those with delayed-pressure urticaria may present with malaise) [7].

## Pathophysiology of urticaria

**Fig. 1:**
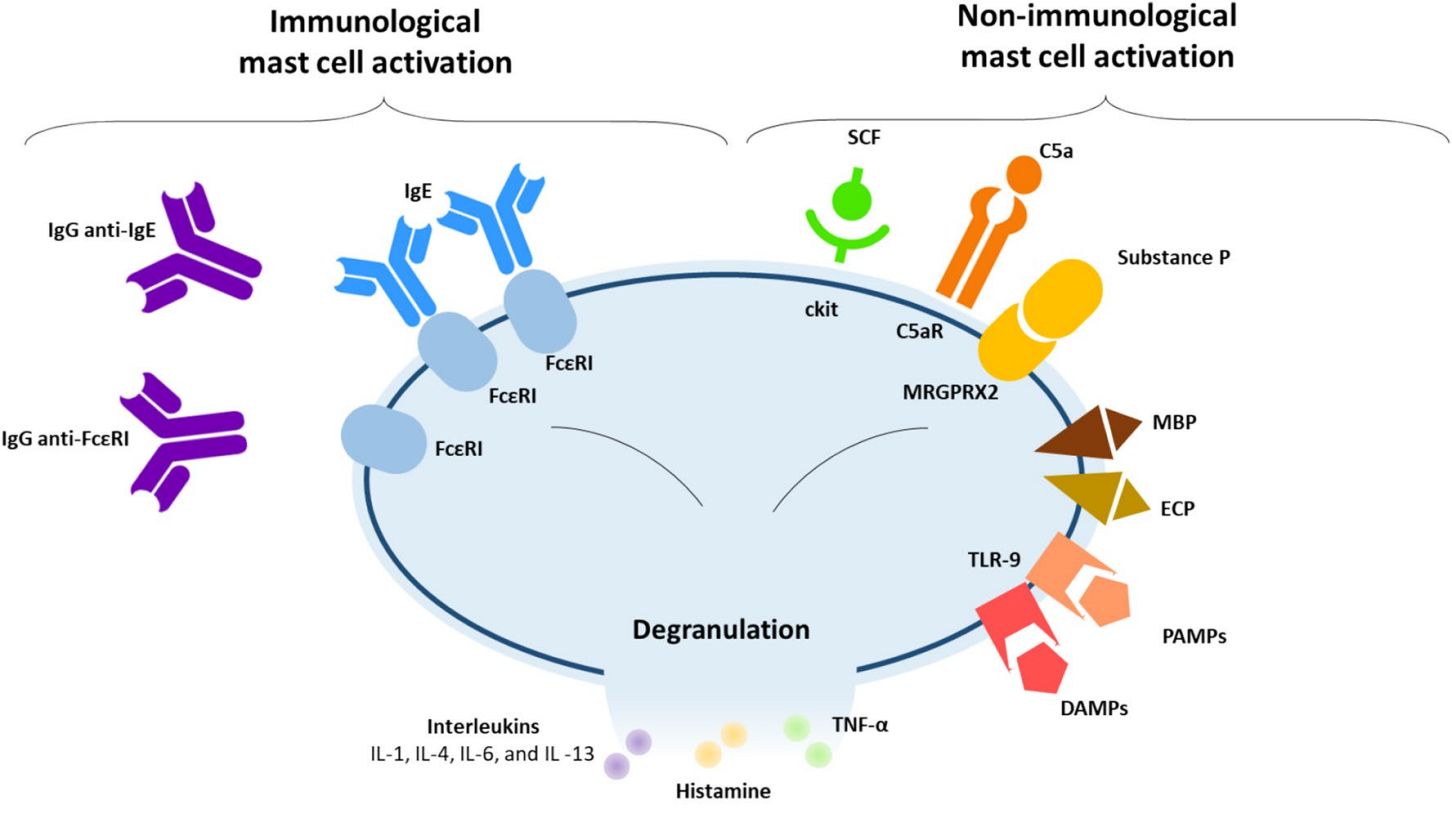
Mast cell activation [6, 10–12]. *DAMPs* damage-associated molecular patterns, *ECP* eosinophil cationic proteins, *FcεRI* high affinity IgE receptor, *IgE* immunoglobulin E, *IgG* immunoglobulin G, *MRGPRX2* mas-related G-protein coupled receptor X2, *MBP* major basic protein, *PAF* platelet activating factor, *PAMPs* pathogen-associated molecular pattern, *SCF* stem cell factor, *TLR* toll-like receptor, *TNF-α* tumor necrosis factor α, *TPO* thyroid peroxidase

Although the exact pathogenesis of urticaria is still poorly understood, investigators agree that urticaria is a mast cell-driven disease resulting from the dysregulation of mast cells and basophils, followed by the release of inflammatory mediators and the stimulation of signaling pathways responsible for the formation of wheals and angioedema [4, 10]. The activation of mast cells is mediated by effectors that are able to interact with membrane receptors (Fig. 1) [6, 10–12].

For example, all mast cells express high-affinity IgE receptors (FcεRI) and are therefore involved in IgE-dependent allergic reactions. When an allergen binds to IgE causing receptor FcεRI cross-linking, the interaction result in mast cell degranulation (type I hypersensitivity or allergy) [13]. Mast cell activation can also occur in the presence of anti-FcεRIα or anti-IgE IgG (type II hypersensitivity or autoimmunity) [14, 15]. Additionally, the participation of IgE autoantibodies directed to autoantigens, e.g., thyroid peroxidase (TPO), double stranded DNA, or interleukin (IL)-24 has also been observed [16–18]. Recent findings seem to support the pathogenic involvement of substance P in urticaria, a neuropeptide associated with the development of vasodilation and pruritus, that further stimulates the activation of mast cells by binding to the membrane mas-related G-protein coupled receptor X2 (MRGPRX2). In chronic urticaria an increase of substance P levels has been reported in apparent correlation with disease severity [19, 20].

**Fig. 2:**
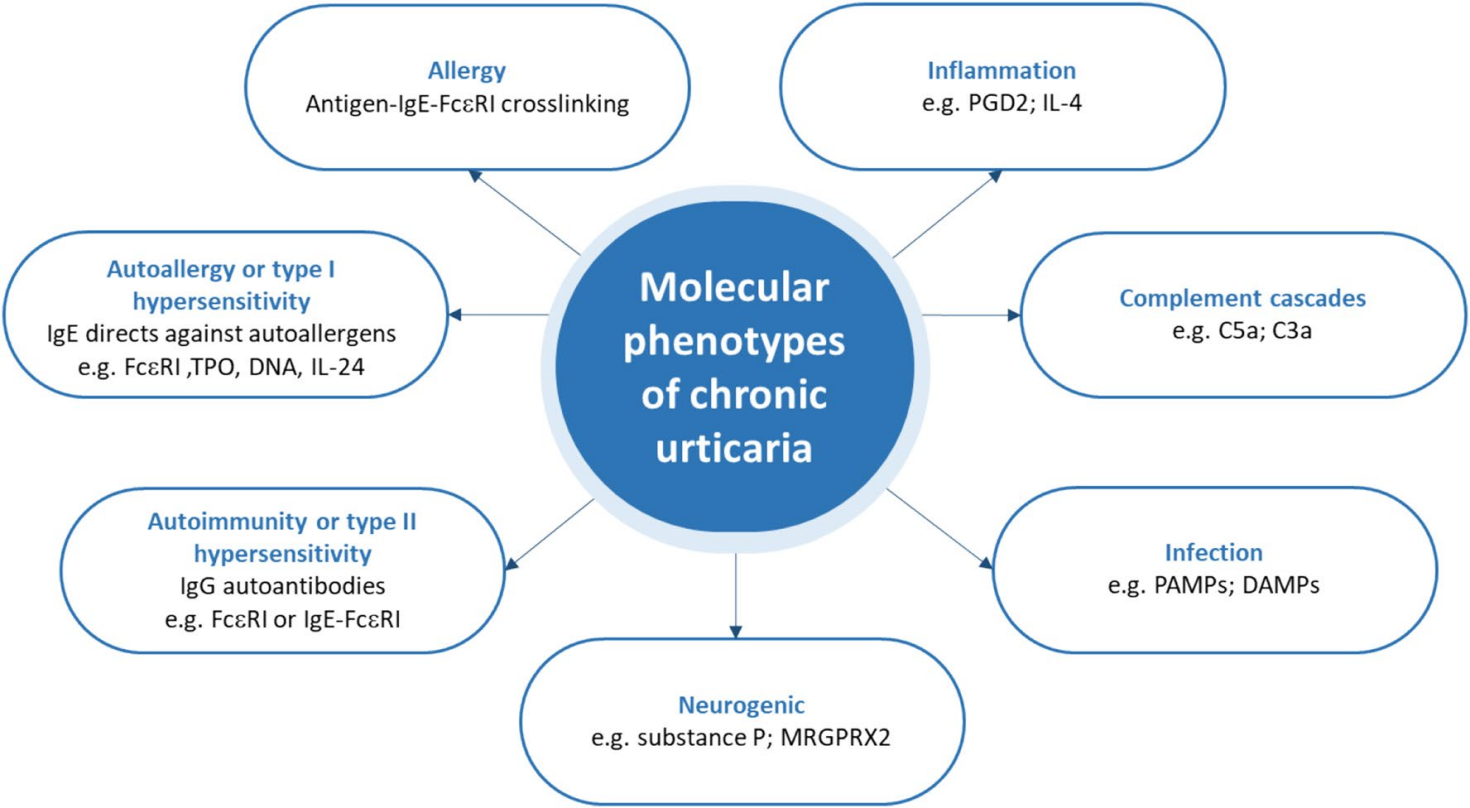
Molecular phenotypes of chronic urticaria [6, 10–12]. *DAMPs* damage-associated molecular patterns, *ECP* eosinophil cationic proteins, *FcεRI* high affinity IgE receptor, *IgE* immunoglobulin E, *IgG* immunoglobulin G, *MRGPRX2* mas-related G-protein coupled receptor X2, *PAF* platelet activating factor, *PAMPs* pathogen-associated molecular pattern, *SCF* stem cell factor, *TPO* thyroid peroxidase

Furthermore, antibodies targeting the eosinophilic receptor CD23 may induce the release of major basic protein (MBP) and eosinophil cationic proteins (ECP), which also activate mast cells [6]. Besides the immunological pathways, there are many other receptors on the mast cell membrane that can also induce cell activation. The complement cascade can activate skin mast cells through its C3a and C5a receptors (C3aR; C5aR) [10]. The interaction between stem cell factor (SCF), a hematopoietic cytokine, and tyrosine kinase receptor c-kit, expressed on the surface of mast cells, promotes the activation, proliferation and development of mast cells [10]. Lastly, different kinds of toll-like receptors (TLRs) recognizing pathogen-associated molecular patterns (PAMPs) and damage-associated molecular patterns (DAMPs) may also be involved [12]. These multiple pathways of mast cell activation and the participation of additional cells and inflammatory compounds explain the observation of distinct clinical phenotypes of urticaria in patients, as shown in Fig. 2 [6, 10–12]. Chronic autoimmune urticaria and inducible urticaria can also be due to underlying mast cell disorders, most commonly secondary or reactive mast cell activation disorders [21]. Advances have been made in understanding the etiological factors of a rare form of urticaria, vibratory urticaria, a condition in which exposing the skin to vibration, repetitive stretching, or friction triggers hives, angioedema, redness erythema, and itching in the affected area. A novel missense substitution in the *ADGRE2* gene, has been identified as the basis of autosomal dominant vibratory urticaria. This substitution results in gain of function in *ADGRE2* which consequently sensitizes mast cells and results in IgE-independent, vibration-induced degranulation [22].

Regardless of the initial factor stimulating mast cell activation, three pathways are consecutively activated. Histamine is released within minutes from mast cell degranulation, together with other preformed inflammatory mediators such as tumor necrosis factor (TNF)-α, IL-1, IL-4, IL-6, and IL-8 [6]. Histamine, TNFα, IL-8, and platelet activating factor (PAF) upregulate the expression of adhesion molecules on endothelial cells and encourage migration of circulating inflammatory cells from the blood into the urticarial lesion [6]. Successively, newly formed cytokines and chemokines are produced by the mast cells. IL-1 and TNF-α recruit leukocytes, particularly eosinophils, neutrophils and T cells inducing the late-phase of the inflammatory response. Lastly, leukotrienes and prostaglandins are produced [6]. Figure 1 summarizes potential ways the mast cell can be induced to undergo degranulation. The release of inflammatory mediators from activated mast cells results in sensory nerve activation, vasodilatation, and plasma extravasation as well as cell recruitment to the urticarial lesions [1, 4].

### Urticaria in COVID-19

The role of infection as a causative factor in multiple forms of urticaria is well documented, thus is not surprising that in the last year reports of different dermatologic manifestations associated with COVID-19 have progressively grown [23]. A heterogeneous pattern of cutaneous manifestations of COVID-19 emerged from recent analysis: vesicular eruption, urticarial eruption, morbilliform eruption, perniolike rash, livedoid rash/retiform purpura, multisystem inflammatory syndrome in children [24]. Although COVID-19 does not have as big an impact on children compared with adults, skin manifestations have been reported also in the pediatric population [25, 26]. Urticarial manifestations accounts for up to 19% of the skin reactions reported and seem to be an early sign of COVID-19, since urticaria can occur hours before other common symptoms appear (cough; dyspnea; fever; fatigue) [24, 27–38]. A recent literature review by Algaadi including 30 articles (202 patients in total) found that more than 50% of COVID-19-associated urticarial rashes were present before or concurrent with classic symptoms of COVID-19 [28]. Recognition that urticaria may be an early sign of severe infection of COVID-19 could prompt interventions to reduce transmission of COVID-19. In the majority of cases, skin lesions have not been associated with COVID-19 severity, although the prospective Spanish cohort study from Galván Casas et al. reported higher morbidity and higher mortality rate for patients with maculopapular lesions [30]. Similarly, in Italy, an analysis conducted on 88 hospitalized patients found that 18 patients (20.4%) developed cutaneous manifestations, either at the onset (n = 8) or after the hospitalization (n = 10) [39]. Since the presence of common urticaria triggers are commonly missing in patients with COVID-19 (e.g. new use or changes in medication) [40], urticaria could be directly related to the pathogenesis of COVID-19. The effect of COVID-19 on the vascular system can occur either directly through cell invasion, or indirectly through inflammation, along with increased levels of leukocytes, cytokines, and chemokines which result in endothelial damage [31]. It is hypothesized that mast cell degranulation is the principal pathophysiological mechanism associated with the systemic organ damage observed in patients with severe forms of COVID-19 [29]. Particularly, the mast cell-induced activation of the kallikrein–kinin system (KKS) could be responsible for the exacerbated inflammatory response observed in COVID-19 patients [41]. KKS is a complex multi-enzyme cascade which produces several active peptides, including bradykinin (BK). The binding of BK, a potent vasodilator and inflammatory mediator, to its receptors (B1R and B2R) triggers the regulation of vascular permeability and inflammatory processes [41]. Of note, most patients with COVID-19 were reported to have elevated levels of circulating IL-6 [42] which has also been associated with the inflammatory response present in some types of urticaria. However, the variety of rashes identified in COVID-19 patients does suggest a possible different pathophysiology, such as the complement activation as shown from the immunohistochemical analysis of some lesions [43]. Angiotensin-converting enzyme (ACE) type II (ACE2) was identified as a functional receptor for the COVID-19 virus and it is thought to play a part in the progression of the infection. Currently, it is known that ACE2 is abundant in human epithelial cells in the lung and small intestine, as well as in the vascular endothelium which then enables easy access to skin cells. Consequently, reactions due to cytokine-induced expression, such as urticarial rashes, may be found among COVID-19 patients [27]. However, the pathogenesis of COVID-19 infection is not yet fully understood and the currently available literature on COVID-19 and urticaria manifestations is based on case reports or incomplete data. Further studies and meta-analysis are required to evaluate the relationship between concurrent urticaria and COVID-19.

## Burden of disease

The disease burden is measured by disability-adjusted life years (DALYs). The 2016 updates from the Global Burden of Disease Study revealed that the global weighted average of DALY rate adjusted for the differences in age distribution among the population (age-standardized DALY) for urticaria is 55.5/100,000 in the general population, 47.4/100,000 in men and 62.5/100,000 in women [44]. Among skin diseases, the burden of urticaria ranks behind acne vulgaris (214/100,000), dermatitis (152/100,000), viral skin disease (80.02/100,000) and psoriasis (76/100,000) only [44]. Chronic urticaria may have a strong impact on patients’ QoL with significant consequences reported on sleep, social interactions and work performance (6% absenteeism) [45]. In addition, individuals suffering from chronic urticaria may develop mental health problems over time, with anxiety and depression reported in more than 30% of patients [45]. The psychological effects of urticaria have been reported separately both in adult and pediatric populations [46–49]. Despite being more frequent in women than men, no marked difference has been shown in the prevalence of mental disorders associated with urticaria symptoms [50].

The economic burden of chronic urticaria is also considerable. Mean yearly direct and indirect costs of chronic urticaria in the United States of North America have been estimated to be $244 million, with medication costs accounting for 62.5%, and work absenteeism for 15.7% of the total cost [51]. The chronic urticaria-related cost has been reported to be as high as $2047 per year per patient in the USA [51]. In France, an economic burden analysis using purchasing power parity dollars (PPP$) demonstrated a high therapy and inpatient cost of almost $3000, whereas in Italy, this cost was $1000. The indirect cost, reported as loss of work productivity, is greater in Germany (≥ PPP$ 1000) than in France (≥ PPP$ 500) [52, 53]. Table 2 summarizes the impact of chronic urticaria on QoL [54].

**Table 2:** Impact of chronic urticaria on patient’s quality of life

Quality of life parameters
1. High costs
2. Presence of comorbidities
3. Unpredictability of symptoms
4. Impact on the family
5. Interference with health-related quality of life
6. School and work decreased performance
7. Resistance to treatment
8. Long disease duration
9. Effects of concomitant angioedema (body deformation, asphyxia)
10. Interference with sexual function
11. Interference with social interactions

Table adapted from Sánchez-Borges et al. [54]

The analysis by age group from the Global Burden of Disease Study showed that children aged 1–9 years old have the highest burden of disease among the pediatric population [44]. Epidemiological data on urticaria in children are limited but the prevalence of all forms of childhood urticaria is estimated to be around 2–6% [9]. Chronic urticaria prevalence varies according to studies. In the UK for instance, the prevalence of chronic urticaria ranges from 0.1% to 0.3% [9]. Acute urticaria is the most common form in childhood with only few cases advancing into a chronic form [3]. In adults, urticaria is more frequent in women than men, but a similar gender difference has not been reported in children [9]. As can be expected, the QoL of children with chronic urticaria is impaired similarly to adults, and daily activities such as school performance, sleep, and social interaction are usually affected [9, 45].

## Diagnosis

Current guidelines for urticaria management recommend a diagnostic work-up that focuses on the examination of clinical signs and assessment of symptoms associated with urticaria [1]. Since urticaria-like lesions can be the manifestation of many other syndromes or skin conditions (lupus erythematosus, dermatomyositis, and polymyositis, Sjögren's syndrome, and Still's disease), a thorough examination assessing frequency, circumstances of onset, duration, local or systemic symptoms is essential to achieve a correct diagnosis [1]. The initial purpose is to discover whether there is any specific trigger of urticaria since the simplest treatment is the avoidance of stimuli. Provocation tests can be performed to confirm inducing factors [1]. In acute urticaria, prick tests or serum specific IgE tests may be helpful to identify an allergen, but only if type I hypersensitivity is suspected based on patient’s clinical history. Skin testing may be difficult in some cases (e.g. dermographism) and is not recommended for those without a history of allergic rhinitis or food allergy. If inducing factors cannot be identified, a complete blood count with differential, erythrocyte sedimentation rate, and/or C-reactive protein is recommended [1]. The autologous skin serum test (ASST) and the histamine-release assay are currently the only available tests for autoimmunity but are not validated to be used diagnostically for autoimmune urticaria [1]. A skin biopsy should be performed in suspected cases of urticarial vasculitis, and if confirmed, systemic vasculitis should be assessed via additional testing [1]. The diagnostic approaches are similar between adult and pediatric populations [3, 9]. Newly developed, validated instruments can quantify chronic spontaneous urticaria severity, control and QoL. These include the urticaria activity score in 7 days (UAS7), the angioedema activity score, the urticaria control test (UCT), the urticaria QoL and angioedema QoL tests.

## Management of urticaria and the role of non-sedating oral antihistamines

The ultimate aim of urticaria treatment is complete symptom control [1]. Although difficult to achieve for some patients, avoidance or elimination of any stimulus or trigger is the first suggested recommendation [1]. Additionally, patients should avoid factors that are known to trigger urticarial symptoms such as alcohol, and if confirmed during the assessment of patient’s clinical history, also, the intake of medications such as aspirin and NSAIDs [1]. When avoidance is not effective, or cannot be achieved, a step-wise pharmacological approach is recommended by several guidelines, as shown in Table 3 [1, 55–58]. Pharmacological treatment recommendations are valid for both adults and children and applicable to all forms of acute and chronic urticaria; on-demand treatment may be more suitable for acute urticaria, whereas chronic inducible urticaria requires continuous treatment [1].

**Table 3:** Summary of clinical guidelines for the treatment of urticaria in adults and children^a^

Therapy	Guidelines				
	EAACI/GA^2^ LEN/ EDF/WAO 2018 [1]	JTF AAAAI/ACAAI 2014 [55]	AADV 2010 [56]	AFP 2014 [57]	JDA 2018 [58]
Step I	Monotherapy with second-generation, non-sedating H_1_-antihistamines^b^	Monotherapy with second-generation H_1_-antihistamines^b^	Avoidance of triggers and physical stimuli	Avoidance of triggers and physical stimuli	Non-sedative second-generation H_1_-antihistamine • As appropriate, change to another drug, increase the dose up to 2 times, or combine the two types
Step II	Up-dosing of non-sedating H_1_-antihistamines^c^ (up to 4 times approved dose; weight adapted)	One or more of the following: • Up-dosing of second-generation H_1_-antihistamines^c^ • Add another second-generation H_1_-antihistamine • Add H_2_-antagonist • Add leukotriene receptor antagonist • Add first generation H_1_-antihistamine	Monotherapy with second-generation, non-sedating H_1_-antihistamines	H_1_-antihistamines: • First generation • Non-sedating second-generation H_2_-antihistamines	Add an alternative agent: • H_2_–antihistamine • Anti-leukotriene
Step III	Add an alternative agent: Omalizumab	Up-dosing of potent antihistamine^c^	Up-dosing of non-sedating H_1_-antihistamines^c^ (up to 4 times approved dose; weight adapted)	• Corticosteroids • Doxepin • Narrowband ultraviolet B light	Add an alternative agent: • Oral corticosteroid • Omalizumab • Cyclosporine
Step IV	Add an alternative agent: • Cyclosporine A	Add an alternative agent: • Omalizumab or cyclosporine • Other anti-inflammatory agents • Immunosuppressants • Biologics	Change to: • Different non-sedating H_1_-antihistamines, or • First generation sedating antihistamine Add an alternative agent: • Leukotriene antagonist	• Cyclosporine	• Trial treatment
Step V	*–*	*–*	Add an alternative agent: *•* Cyclosporine A *•* Second-generation, non-sedating H_2_-antihistamine *•* Dapsone *•* Omalizumab	*•* Dapsone, *•* Intravenous immunoglobulin *•* Methotrexate	*–*

*JTF AAAAI* Joint task force of the American Academy of Allergy, Asthma & Immunology, *AADV* Asian Academy of Dermatology and Venereology; *ACAAI* American College of Allergy, Asthma & Immunology, *AFP* Australian Family Physician, *EAACI* European Academy of Allergology and Clinical Immunology, *EDF* European Dermatology Forum, *GA*^*2*^*LEN* Global Allergy and Asthma European Network, *JDA* Japanese Dermatological Association, *WAO* World Allergy Organization

^a^An interval of 2 to 4 weeks should be allowed before changing therapy

^b^Avoidance of triggers and physical stimuli is considered only as commentary and not included in the step-wise approach

^c^Off-label use

Second-generation non-sedating H_1_-antihistamines are the cornerstone of first-line treatment [1]. There is a strong recommendation against the long-term use of oral glucocorticoids [1], however, in severe cases, short courses of glucocorticoids can be used up to a maximum of 10 days to control symptoms [1]. Although the same algorithm for adults is applicable for a pediatric population, only medications with proven efficacy and safety should be used in children, and options may vary across countries [1]. Similar recommendations are applicable to women who are pregnant or lactating [1]. The efficacy and safety of bilastine, cetirizine, desloratadine, fexofenadine HCl, levocetirizine, loratadine, mizolastine (> 12 years old) and rupatadine have been well established in the pediatric population. However, treatment should be based on individual considerations and taken cautiously as data on the efficacy in children are limited [1].

Patients unresponsive to antihistamines should be referred to a clinical specialist with expertise in the evaluation and management of urticaria and/or angioedema. The treatment of patients unresponsive to second-generation H_1_-antihistamines varies depending on local regulation and approved medicines. The European Academy of Allergology and Clinical Immunology, the Global Allergy and Asthma European Network, the European Dermatology Forum and the World Allergy Organization (EAACI/GA^2^LEN/ EDF/WAO) 2018 guidelines recommend the use of omalizumab, a monoclonal anti-IgE antibody, for the treatment of unresponsive chronic urticaria [1, 21]. Ciclosporin A also showed a moderate, direct effect on mast cell mediator release in placebo-controlled trials, however, its use is mostly off-label due to safety concerns and is recommended only for patients with severe disease refractory to any dose of antihistamine and omalizumab in combination [1]. Lastly, the use of leukotriene antagonists (e.g. montelukast), sulphasalazine, methotrexate, interferon, plasmapheresis, phototherapy and intravenous immunoglobulins could be considered under the control of a specialist, but they are not currently recommended by the EAACI/GA^2^LEN/ EDF/WAO 2018 guidelines due to lack of evidence [1].

The classical recommended approach for the management of urticaria with first-line H_1-_antihistamines alone has been proven to be inadequate to stop mast cell histamine degranulation in COVID-19 patients, but it can reduce the severity of urticaria in some patients [29, 59]. Antihistamines in combination with low doses of systemic glucocorticoids may also improve the clinical response of COVID-19 patients with urticaria. Glucocorticoids and immunosuppressants should be cautiously considered to avoid impairment of the T-cell mediated immune response to the virus.

## Oral H_1_-antihistamines

Histamine and its four receptors (H_1_R–H_4_R) are important mediators of the immune response and allergic inflammation; the H1-receptor drives cellular migration, nociception, and vasodilatation [60]. H_1_-antihistamines act as inverse agonists by stabilizing the H_1_-receptor in its inactive conformation, therefore preventing normal functioning [60]. H_1_-antihistamines down-regulate allergic inflammation via direct or indirect down-regulation of antigen presentation, expression of pro-inflammatory cytokines and cell adhesion molecules, and chemotaxis of inflammatory effector cells [60]. H_1_-antihistamines are categorized as first- and second-generation antihistamines. First generation H_1_-antihistamines are less selective than second-generation as interaction with other types of receptors has been extensively reported (muscarinic, α-adrenergic, serotonin receptors). Because of this limited receptor selectivity, adverse effects such as paradoxical excitation, irritability, hyperactivity and hallucinations, constipation, dry mouth, urinary retention, and tachycardia have been reported [60, 61]. Additionally, first generation H_1_-antihistamines can cross the blood–brain barrier and act on H_1_-receptors in the central nervous system which can interfere with histaminergic neurotransmission, thereby causing drowsiness, sedation, somnolence, fatigue, and headache [61]. First generation H_1_-antihistamines have been associated with impairment of cognitive function, memory, and psychomotor performance. Global guidelines strongly recommend against the use of first generation H_1_-antihistamines due to their adverse effects; their use is only recommended in countries where second-generation H_1_-antihistamines are not available (mainly South-East Asian countries) or when maximum doses of second-generation antihistamines are ineffective. However, when the use of first-generation antihistamines is necessary, possible benefits and risks should be thoroughly discussed with the patient before making any decision [1]. Second-generation H_1_-antihistamines were developed to be devoid of such adverse effects, some of them are selective inverse agonists for H_1_-receptor which do not cross the blood brain barrier, resulting in minimal or non-sedating effect [61]. Second-generation H_1_-antihistamines include acrivastine, bilastine, cetirizine, desloratadine, fexofenadine HCl, levocetirizine, loratadine, and mizolastine, although several of these agents are not available in all countries [3]. Among these, fexofenadine HCl, bilastine, desloratadine and levocetirizine do not require hepatic metabolism to be active, in contrast to the other second-generation H_1_-antihistamines. It is beyond the scope of this review to discuss all second-generation antihistamines, and fexofenadine HCl is presented as representative of the effectiveness of this class of medicines for the treatment of urticaria. Fexofenadine is a non-sedating second generation commercialized for 25 years in more than 100 countries around the world [62].

## Fexofenadine HCl for the treatment of urticaria in adults and children

As a second-generation, non-sedating antihistamine, fexofenadine HCl has been widely used in allergic diseases and is available as an oral tablet, or liquid suspension for the control of urticaria symptoms. The approved dose for the treatment of chronic urticaria is oral tablet 180 mg once a day or 60 mg orally 2 times a day both in adults and children 12 years and older. Fexofenadine oral suspension 15 or 30 mg (according to age) twice a day is available for children older than 6 months [62].

## Therapeutic efficacy of fexofenadine HCl

The most common method used to assess blockade of histamine H_1_–receptors is inhibition of the histamine-induced wheal and flare via skin tests (histamine test). This technique provides objective data about the onset, potency and duration of action of antihistamines, although its predictive value on drug effectiveness needs to be confirmed [63].

When directly compared with placebo, fexofenadine HCl 60, 120, 180 or 240 mg suppressed histamine-induced wheal and flare reactions. A recent meta-analysis performed by Huang et al., found that antihistamine effect of fexofenadine HCl was significantly higher than that of placebo (p < 0.00001, both flare and wheal), and non-inferior to other second-generation antihistamines (flare, p = 0.84; wheal, p = 0.21) based on the analysis of five studies on healthy subjects [64]. In a recent study conducted in Brazil, 10 healthy adults were subjected to the histamine test to compare the effect of the H_1_-antihistamines most used in the local clinical practice (dexchlorpheniramine, hydroxyzine, levocetirizine, fexofenadine HCl, cetirizine, loratadine, ebastine, desloratadine, epinastine and rupatadine) [65]. Two hours after intake, all antihistamines including fexofenadine HCl resulted in a significant reduction in the wheal (p < 0.02) as well as in the flare compared to control [65]. Fexofenadine HCl 180 mg compared with desloratadine 5 mg significantly reduced histamine-induced flares (61% versus + 2%, respectively: p < 0.05) and wheals (p < 0.05) at 2 h after treatment in adults and adolescents [66]. Other studies of H_1_-antihistamines with slightly different methodologies confirmed similar results regarding the efficacy of fexofenadine HCl in the suppression of the induced wheal [67, 68].

Well-designed clinical trials have shown the efficacy of fexofenadine in patients suffering from urticaria. In a multicenter, double-blind study conducted by Paul et al., the reduction of mean daily total symptom score (TSS) of pruritus and wheals was found to be dosage-dependent and statistically significant compared with placebo for fexofenadine HCl 180 mg (p = 0.0041) and 240 mg once-a-day (p = 0.0008), but not for fexofenadine HCl 60 or 120 mg once-a-day [69]. However, mean daily TSS in the combined group receiving fexofenadine HCl was significantly improved compared with placebo (p = 0.0019) [69]. Similar findings were reported in the pediatric population. A randomized, placebo-controlled study enrolling 163 patients (> 12 years old) evaluated the mean daily number of wheals and the mean daily severity of pruritus during 180 mg fexofenadine HCl treatment. After a 4-week treatment period, fexofenadine HCl showed greater and significant improvements in both endpoints compared with placebo (mean change in daily number wheals: fexofenadine HCl, − 0.78; placebo, − 0.40; mean change in mean pruritus severity: fexofenadine HCl, − 1.04; placebo, − 0.57; p < 0.001 both) [70]. An observational study conducted in Egypt showed that fexofenadine HCl significantly improved all signs of chronic urticaria after 4 week of treatment; by the end of the study, pruritus and hives status was completely relieved in the majority of participants (74.9% and 81.1%, respectively; p < 0.001) [71].

Up-dosing of antihistamines up to fourfold the licensed dose is recommended by the major clinical guidelines for the treatment of urticaria if control is not achieved, although the indication is based on the expert’s opinion. A recent systematic review assessing the results of 14 studies, of which six were placebo-controlled randomized trials, found that increasing doses of fexofenadine HCl (up to 720 mg; off-label dosage) resulted in better control of urticaria symptoms with minor treatment-related adverse events (headache) [72]. It has been shown that treatment with fexofenadine HCl improves health-related QoL and does not interfere with work productivity or performance of daily activities in patients with chronic urticaria [73, 74]. Table 4 summarizes the studies investigating the efficacy and QoL of fexofenadine HCl [65–71, 73–76].

**Table 4:** Summary of the studies investigating the efficacy of fexofenadine HCl in pediatric and adult populations

Study	Treatment and dosage	Patient population, N	Fexofenadine HCl outcomes
Maciel-Guerra et al. 2018 [65]	Dexchlorpheniramine 2 mg, hydroxyzine 25 mg, levocetirizine 5 mg, fexofenadine HCl 180 mg, cetirizine 10 mg, loratadine 10 mg, ebastine 10 mg, desloratadine 5 mg, epinastine 20 mg, rupatadine 10 mg	Adults (healthy), N = 10	• All antihistamines suppressed wheal (p < 0.02) and flares, except for rupatadine (p = 0.70) in histamine test
Meltzer et al. 2007 [66]	Fexofenadine HCl, 180 mg, desloratadine, 5 mg or placebo	Adolescents ^a^ and adults (healthy), N = 54	• Fexofenadine HCl was significantly superior to desloratadine in the suppression of wheal (at 2–6 h, p ≤ 0.005) in histamine test. Fexofenadine was also superior to in suppression of flares 3 h (83% vs 18%, respectively), 4 h (79% vs 3%, respectively), 5 h (75% vs 27%, respectively) and 6 h (85% vs 36%, respectively) post-treatment (p < 0.05) in histamine test
Tanizaki et al. 2012 [67]	Bepotastine besilate 10 mg; fexofenadine HCl 60 mg; or placebo	Adults (healthy), N = 10	• Fexofenadine HCl suppressed wheal and flare 3 h after histamine test (p < 0.05), and itch within 30 min (p < 0.05)
Purohit et al. 2004 [68]	Fexofenadine HCl, 180 mg; or cetirizine 10 mg	Adults (healthy), N = 42	• Frequency of 95% or greater wheal inhibition occurred with fexofenadine at 1.5–2.5 h compared with 3–4 h with cetirizine, but this difference was not statistically significant
Paul et al. 1998 [69]	Fexofenadine HCl 60, 120, 180, 240 mg QD; or placebo	Adults (urticaria), N = 222	• Significant reduction of mean daily TSS in the combined fexofenadine HCl group compared with placebo (73–81% vs 54%, respectively, p = 0.0019) • The response was dose-dependent (p = 0.001)
Fouad et al. 2017[71]	Fexofenadine HCl (details not available)	Adults (urticaria), N = 498	• At the end of the study 74.9% and 81.1% of patients had pruritus and hives cured, respectively (p < 0.001, both)
Kaplan et al. 2005 [70]	Fexofenadine HCl, 180 mg QD; or placebo	Adolescents ^a^ and adults (urticaria), N = 255	• Reduction of mean daily wheal score (fexofenadine HCl, − 0.78; placebo, − 0.40) and mean daily pruritus score (fexofenadine HCl, − 1.04; placebo, − 0.57); p < 0.001 both
Nelson et al. 2000 [75]	Fexofenadine HCl 20, 60, 120, 240 mg BID; or placebo	Adolescents ^a^ and adults (urticaria), N = 418	• Mean (SE) change in pruritus score at week 4 was: − 0.68 (0.10); − 1.12 (0.09); − 0.87 (0.10); − 1.15 (0.10) for fexofenadine HCl 20, 60, 120 and 240 mg, respectively (p = 0.0019) • All doses were superior in reducing interference with sleep (p ≤ 0.0011) and daily activities than placebo (p ≤ 0.0014)
Finn et al. 1999 [76]	Fexofenadine HCl 20, 60, 120, or 240 mg BID; or placebo	Adults (urticaria), N = 439	• Mean (SE) change in pruritus score at week 4 was: − 1.17 (0.08); − 1.15(0.08); − 1.13(0.08); − 1.29(0.08) for fexofenadine HCl 20, 60, 120 and 240 mg, respectively (p = 0.0001) • All doses reduced the mean number of wheals score compared with placebo (p ≤ 0.0238); improvement was dose-related (p = 0.0001)
Thompson et al. 2000 [73]	Fexofenadine HCl 60 mg BID; placebo	Adolescents ^a^ and adults (urticaria), N = 325	• Fexofenadine improved DLQI score from baseline compared with placebo (10.0−10.6 vs 11.0–12.1, respectively; p ≤ 0.0002) • Improved overall productivity at work (9.5–7.0% higher than placebo; p ≤ 0.152), in the classroom productivity (not significant), and in regular activities (10.2–10.0% higher than placebo; p ≤ 0.0002)
Spector et al. 2007 [74]	Fexofenadine HCl 180 mg QD, or placebo	Adults (urticaria), N = 254	• Improvements in mean total DLQI score (p = 0.0219) compared with placebo • Less impairment in work productivity, overall work, and activity

*BID* twice daily, *DLQI* Dermatology Life Quality Index, *SE* standard error, *QD* once daily, *QoL* quality of life, *TSS* total symptom scores

^a^ ≥ 12 years old

## Safety of fexofenadine HCl

Overall, fexofenadine HCl is well-tolerated and discontinuation due to adverse effects generally occurs in less than 5% of patients [64].

In adults, second-generation antihistamines are not considered to be cardiotoxic (e.g. potassium channel blockade or QT interval prolongation) [77]. In healthy individuals, fexofenadine HCl did not prolong QTc or decrease heart rate [78]. Prolongation of QTc occurs through blockade of potassium channels in ventricular myocytes, leading to a delay in ventricular repolarization; fexofenadine HCl does not appear to block this channel [78]. Clinical studies have further confirmed the cardiovascular safety of fexofenadine HCl [64]. Fexofenadine HCl is devoid of central nervous system effects [79, 80]. Hiraoka et al. reported a lower sedative effect of fexofenadine HCl than most first and second-generation antihistamines since it does not cross the blood–brain barrier [81]. The effect of second-generation antihistamines on cognitive function was evaluated on eighty-eight patients with mild and moderate chronic urticaria (16–53 years old) receiving cetirizine, levocetirizine, fexofenadine HCl, ebastine and desloratadine for one month. Cognitive functions were assessed by the attention test and the activity of thought test. Fexofenadine HCl, desloratadine and ebastine had no effect on the attention or thought processes of patients when compared with impairments induced by other antihistamines [82]. Additionally, with fexofenadine HCl there was no cognitive/psychomotor impairment whereas there was evidence of some impairment with other first- and second-generation antihistamines [64]. A recent systematic review ranked fexofenadine HCl as having the least psychomotor impairment induced by antihistamines compared with all other antihistamines on the Japanese market [83]. The safety profile of fexofenadine HCl is also corroborated in that it does not interfere with driving performance, as demonstrated in a double-blind, randomized, placebo-controlled, crossover study, where healthy volunteers were evaluated for cognitive performance while performing simulated driving tasks. Participants received either 60 mg fexofenadine, 5 mg levocetirizine, 50 mg diphenhydramine as a positive control, or placebo. Both antihistamines did not impair the performance of car-driving tasks. Fexofenadine HCl showed no significant psychomotor difference compared with placebo (p < 0.03) across tests [84]. In another randomized, placebo-controlled crossover study, forty-two healthy naval aviation personnel were evaluated for subjective drowsiness, cognitive performance, and vigilance after receiving either 180 mg fexofenadine or 50 mg diphenhydramine as a positive control, or placebo. Diphenhydramine administration resulted in significant psychomotor decrements compared with fexofenadine, whereas the effects of fexofenadine were similar to placebo. Subjects performed faster and better with fexofenadine vs. diphenhydramine on measures of omission errors and commission errors (p = 0.05) [85].

In children, only a few H_1_-antihistamines have been investigated for safety: cetirizine, levocetirizine, loratadine, fexofenadine HCl, desloratadine, and rupatadine [86, 87]. A study conducted on children aged 2–5 years with allergic rhinitis receiving fexofenadine HCl 30 mg, showed a good safety profile and tolerability [88].

Fexofenadine HCl is considered well tolerated in adults and children, and safe to take if necessary during pregnancy or while lactating, however it is recommended to follow the fexofenadine prescribing information [61, 64]. Table 5 summarizes the studies investigating the safety of fexofenadine HCl [44, 78–80, 84, 88–92].

**Table 5:** Summaries of the studies investigating the safety of fexofenadine HCl in pediatric and adult populations

Study	Treatment and dosage	Treatment duration/ time of analysis post administration	Patient population, N	Fexofenadine HCl outcomes
Pratt et al. 1999 [78]	Fexofenadine HCl 60, or 80 mg BID; fexofenadine HCl 240 mg QD; placebo	3 up to 12 months	Adults, N = 930	• No significant increases in QTc between fexofenadine and placebo (p ≥ 0.188)
Bernstein et al. 1997 [89]	Fexofenadine HCl 60, 120, or 240 mg BID; or placebo	14 days	Adults, N = 570	• No significant difference in treatment-related AEs were noted between fexofenadine (10.9%) and placebo (9.2%) • No sedative effects or ECGs abnormalities, including prolongations in QTc
Hindmarch et al. 1999 [79]	Fexofenadine HCl 80, 120 and 180 mg, loratadine 10 mg, promethazine 30 mg, and placebo	Up to 24 h (post administration)	Adults (healthy), N = 24	• No disruptive effects on aspects of psychomotor and cognitive function
Hindmarch et al. 2002 [80]	Fexofenadine HCl 360 mg, promethazine 30 mg and placebo	7 h (post administration)	Adults (healthy), N = 15	• No disruptive effects on aspects of psychomotor and cognitive function
Hampel et al. 2003 [90]	Fexofenadine HCl 180 mg, cetirizine 10 mg	2 weeks (post administration)	Adults, N = 495	• Fexofenadine resulted in significantly less overall drowsiness vs baseline than those receiving cetirizine (− 2.33 [95% CI, − 3.80 to 0.86] vs. 0.37 [95% CI, − 1.10 to 1.84]; p = 0.0110)
Inami et al. 2016 [84]	Fexofenadine HCl 60 mg; levocetirizine 5 mg; diphenhydramine 50 mg; placebo	90 and 180 min (post-administration)	Adults, N = 20	• No significant difference compared with placebo on psychomotor performance (p > 0.03)
Bower et al. 2003 [85]	Fexofenadine HCl 180 mg; diphenhydramine 50 mg or placebo	90 min (post-administration)	Adults, N = 42	• Effects of fexofenadine on psychomotor performance were similar to placebo. No AE reported with fexofenadine 180 mg
Milgrom et al. 2007 [88]	Fexofenadine HCl 30 mg BID, or placebo	2 weeks	Children (2–5 years old), N = 453	• AEs possibly related to treatment were experienced by 19 (8.2%) and 21 (9.5%) of participants receiving placebo and fexofenadine, respectively. No clinically relevant differences were found in ECGs, laboratory measures, vital signs, or physical examination results compared to placebo • No prolongation in QT interval was observed (fexofenadine: − 0.8 [17.2]; placebo: 4.1 [19.9])
Meltzer et al. 2004 [91]	Fexofenadine HCl 15; 30 or 60 mg BID; or placebo	2 weeks	Children (6–11 years old), N = 1810	• Most common reported AE was headache (4.3% placebo; 7.2% fexofenadine [any dose]) • Adverse events were similar across treatment groups (24.4% placebo; 24.1% fexofenadine [30 mg BID]; 28.4% fexofenadine [any dose])
Segall et al. 2008 [92]	Fexofenadine HCl 30 mg; placebo	Up to 24 h (post administration)	Children (2–5 years old), N = 50	• No trends or clinically meaningful changes in mean ECG, vital sign, or clinical laboratory test data occurred
Maciel-Guerra et al. 2018 [65]	Dexchlorpheniramine 2 mg, hydroxyzine 25 mg, levocetirizine 5 mg, fexofenadine HCl 180 mg, cetirizine 10 mg, loratadine 10 mg, ebastine 10 mg, desloratadine 5 mg, epinastine 20 mg, rupatadine 10 mg	2 h post administration	Adults (healthy), N = 10	• Drowsiness was reported with dexchlorpheniramine (60%), hydroxyzine (80%), levocetirizine (30%), cetirizine (40%), loratadine (20%), ebastine (20%), desloratadine (10%), epinastine (20%), and rupatadine (30%), but not with fexofenadine (0%)

*AE* adverse events, *ECG* electrocardiogram

## Conclusion

The burden of acute and chronic urticaria is high, in terms of patient quality of life and disability-adjusted life years. The aim of treatment for urticaria is that of full control of the associated wheals and angioedema, and second generation anti-histamines such as fexofenadine HCl have been shown to significantly reduce these symptoms whilst being well-tolerated. Recently, urticaria has been reported as a manifestation of COVID-19, however further analyses are needed to fully establish the link.

## Data Availability

Not applicable.

## Abbreviations

AADV: Asian Academy of Dermatology and Venereology
ACAAI: American College of Allergy, Asthma & Immunology
ACE: Angiotensin-converting enzyme
AE: Adverse events
AFP: Australian family physician
ASST: Autologous skin serum test
BID: Twice daily
COVID-19: Coronavirus disease 2019
DALY: Disability-adjusted life years
DAMP: Damage-associated molecular patterns
DLQI: Dermatology Life Quality Index
EAACI: European Academy of Allergology and Clinical Immunology
ECG: Electrocardiogram
ECP: Eosinophil cationic proteins
EDF: European Dermatology Forum
FcεRI: High affinity immunoglobulin-E receptor
GA^2^LEN: Global Allergy and Asthma European Network
H: Histamine
HCl: Hydrochloride
HR: Histamine receptor
Ig: Immunoglobulin
IL: Interleukin
JDA: Japanese Dermatological Association
JTF AAAAI: Joint task force of the American Academy of Allergy, Asthma & Immunology
MBP: Major basic protein
MRGPRX2: Mas-related G-protein coupled receptor X2
NSAIDs: Non-steroidal anti-inflammatory drugs
PAF: Platelet activating factor
PAMP: Pathogen-associated molecular patterns
QD: Once daily
QoL: Quality of life
SCF: Stem cell factor
SE: Standard error
TLR: Toll-like receptor
TNF: Tumor necrosis factor
TPO: Thyroid peroxidase
TSS: Total symptom score
UAS7: Urticaria activity score in 7 days
UCT: Urticaria control test
WAO: World Allergy Organization
