# Correction to: Insights into urticaria in pediatric and adult populations and its management with fexofenadine hydrochloride

**DOI:** 10.1186/s13223-022-00705-y

**Published:** 2022-07-23

**Authors:** Ignacio J. Ansotegui, Jonathan A. Bernstein, Giorgio W. Canonica, Sandra N. Gonzalez-Diaz, Bryan L. Martin, Mario Morais-Almeida, Margarita Murrieta-Aguttes, Mario Sanchez Borges

**Affiliations:** 1 Department of Allergy and Immunology, Hospital Quironsalud Bizkaia, Leioa-Unbe Errepidea, 33 Bis, Erandio, 48950 Bilbao, Spain; 2 Department of Internal Medicine, Division of Allergy and Immunology, University of Cincinnati, Cincinnati, OH USA; 3 Personalized Medicine, Asthma and Allergy, Humanitas University and Research Hospital, Rozzano, Milan Italy; 4 Regional Center for Allergy and Clinical Immunology, Universidad Autónoma de Nuevo León, Monterrey, México; 5 Medicine and Pediatrics, The Ohio State University in Columbus, Columbus, OH USA; 6 Allergy Center, CUF Descobertas Hospital, CUF Academic and Research Medical Center, Lisbon, Portugal; 7 Sanofi, Scientific Innovation, Gentilly, France; 8 Allergy and Clinical Immunology Department, Centro Médico Docente La Trinidad, Caracas, Venezuela; 9 Department of Biomedical Sciences, Humanitas University, Pieve Emanuele, MI Italy

## Correction to: Allergy, Asthma & Clinical Immunology (2022) 18:41 https://doi.org/10.1186/s13223-022-00677-z

In this article [1], the affiliation 'Department of Biomedical Sciences—Humanitas University—Pieve Emanuele (MI), Italy' for Giorgio W. Canonica was missing. It has been corrected with this correction.
